# Hare’s Text-Book of Practical Therapeutics: A Text Book of Practical Therapeutics; with Especial Reference to the Application of Remedial Measures to Disease and Their Employment upon a Rational Basis

**Published:** 1894-10

**Authors:** 


HARE'S TEXT-BOOK OF PRACTICAL THERAPEUTICS: A TEXT-BOOK OF PRACTICAL THERAPEUTICS; WITH ESPECIAL REFERENCE TO THE APPLICATION OF REMEDIAL MEASURES TO DISEASE AND THEIR EMPLOYMENT UPON A RATIONAL BASIS. By Hobart Amory Hare, M.D., Professor of Therapeutics and Materia Medica in the Jefferson Medical College of Philadelphia. With special chapters by Drs. G. E. de Schweinitz, Edward Martin, and Barton C. Hirst. New (fourth) Edition, thoroughly Revised and much Enlarged. In one Octavo Volume of 740 Pages. Cloth, $3.75; Leather, $4.75.

As a text-book for medical students and a handy reference book for the practitioner, we know of no work its superior, if, indeed, it has an equal. Divested of abstract materia medica, it gives clearly and concisely the application of drugs to disease. Any attempt at grouping drugs by classification has been abandoned and the drugs are arranged alphabetically, thus enabling one to find a name easily without consulting the index, though this is complete and well made. The new remedies of merit, whether in the pharmacopeia or not, are fully discussed, and the student will find an impartial and scientific exposition of the merits and shortcomings of each drug treated of.

In addition to the description and action of drugs, the latter half of the work, devoted to diseases, goes fully into the rational therapeutics and application of remedies. The book is without a peer in its sphere.

---

INTERNATIONAL CLINICS, Volume II., Fourth Series. By Lippincott & Co.

The *International Clinics* are so well and favorably known to the profession that more than a passing notice is scarcely necessary. The present volume is rich in matter and illustrations, and contains articles from the pens of the leading physicians of both America and Europe. The *Clinics* constitute a library within themselves, setting forth the best medical opinion of the day in both surgery and general medicine, and no physician who desires to know what is taking place in the medical world can well afford to do without them.

